# Construction of a prognostic model related to copper dependence in breast cancer by single-cell sequencing analysis

**DOI:** 10.3389/fgene.2022.949852

**Published:** 2022-08-23

**Authors:** Xiao Guan, Na Lu, Jianping Zhang

**Affiliations:** Department of General Surgery, The Second Affiliated Hospital of Nanjing Medical University, Nanjing, Jiangsu, China

**Keywords:** breast cancer, copper dependence, prognostic model, bioinformatics, single-cell sequencing analysis

## Abstract

**Purpose:** To explore the clinical significance of copper-dependent-related genes (CDRG) in female breast cancer (BC).

**Methods:** CDRG were obtained by single-cell analysis of the GSE168410 dataset in the Gene Expression Omnibus (GEO) database. According to a 1:1 ratio, the Cancer Genome Atlas (TCGA) cohort was separated into a training and a test cohort randomly. Based on the training cohort, the prognostic model was built using COX and Lasso regression. The test cohort was used to validate the model. The GSE20685 dataset and GSE20711 dataset were used as two external validation cohorts to further validate the prognostic model. According to the median risk score, patients were classified as high-risk or low-risk. Survival analysis, immune microenvironment analysis, drug sensitivity analysis, and nomogram analysis were used to evaluate the clinical importance of this prognostic model.

**Results:** 384 CDRG were obtained by single-cell analysis. According to the prognostic model, patients were classified as high-risk or low-risk in both cohorts. The high-risk group had a significantly worse prognosis. The area under the curve (AUC) of the model was around 0.7 in the four cohorts. The immunological microenvironment was examined for a possible link between risk score and immune cell infiltration. Veliparib, Selumetinib, Entinostat, and Palbociclib were found to be more sensitive medications for the high-risk group after drug sensitivity analysis.

**Conclusion:** Our CDRG-based prognostic model can aid in the prediction of prognosis and treatment of BC patients.

## Introduction

As the most common malignancy, BC accounts for 15.5% of female cancer deaths worldwide, which poses a heavy burden on global health (Sung et al., 2021). Based on genomic and transcriptomic sequences, BC can be classified into five molecular subtypes (Rainey et al., 2020). However, due to the heterogeneity of BC, although multiple prognostic tools have been developed, none of them can predict the prognosis of all types of BC (Krop et al., 2017; Faria et al., 2021). Moreover, the overall prognosis of BC is poor, especially for advanced patients (Tao et al., 2015). Advanced BC with distant organ metastases is considered incurable (Harbeck et al., 2019). Therefore, finding novel prognostic factors and treatment targets for BC to guide clinical practice is critical.

Multiple cells in BC now can be studied accurately due to the advances in single-cell sequencing, which is a strong method for characterizing diverse cell types, and has been used to study a variety of cancers (Treutlein et al., 2016; Ziegenhain et al., 2017). At the same time, through cell clustering and annotation, we can understand the cellular differentiation and immune mechanisms of BC better (Hwang et al., 2018). Defects in the execution of cell death by tumor cells are one of the main reasons for their resistance to therapy (Hassannia et al., 2019).

As a form of regulated cell death, copper-dependent death occurs through the direct binding of copper to fatty acylation components of the tricarboxylic acid cycle (TCA) (Tsvetkov et al., 2022). Copper has two roles in carcinogenesis: it promotes tumor development while also causing redox stress in cancer cells (Maung et al., 2021). High levels of copper promote drug resistance and repair of damaged DNA in cancer cells through the induction of *MDC1* expression by copper chaperones (Jin et al., 2022). It has been shown that reducing copper uptake by knocking out human copper transporter protein 1 can inhibit prostate cancer cell proliferation and tumor growth (Xie and Peng, 2021). The study by *Teng et al.* also confirmed that copper deficiency may be a novel approach to the treatment of pancreatic cancer (Yu et al., 2019). Besides, copper can also regulate proteins involved in evading immune responses, such as the transmembrane protein programmed death ligand 1. Interaction between PD-L1 and PD-1 receptors on cytotoxic T lymphocytes prevents immune cells from attacking cancer cells (Voli et al., 2020). Therefore, limiting the availability of copper during carcinogenesis may be one way to slow cancer progression (Shanbhag et al., 2021). It is crucial to explore the role of CDRG in cancer. Nevertheless, whether these CDRG are associated with the prognosis of BC patients is uncertain.

Herein, we first identified CDRG in BC by single-cell sequencing. Based on these CDRG, we constructed a prognostic model which could evaluate the prognosis of BC patients accurately. At the same time, the immune microenvironment and medication sensitivity of BC are likewise linked to CDRG. This study informed the treatment strategy for BC.

## Methods

**FIGURE 1:**
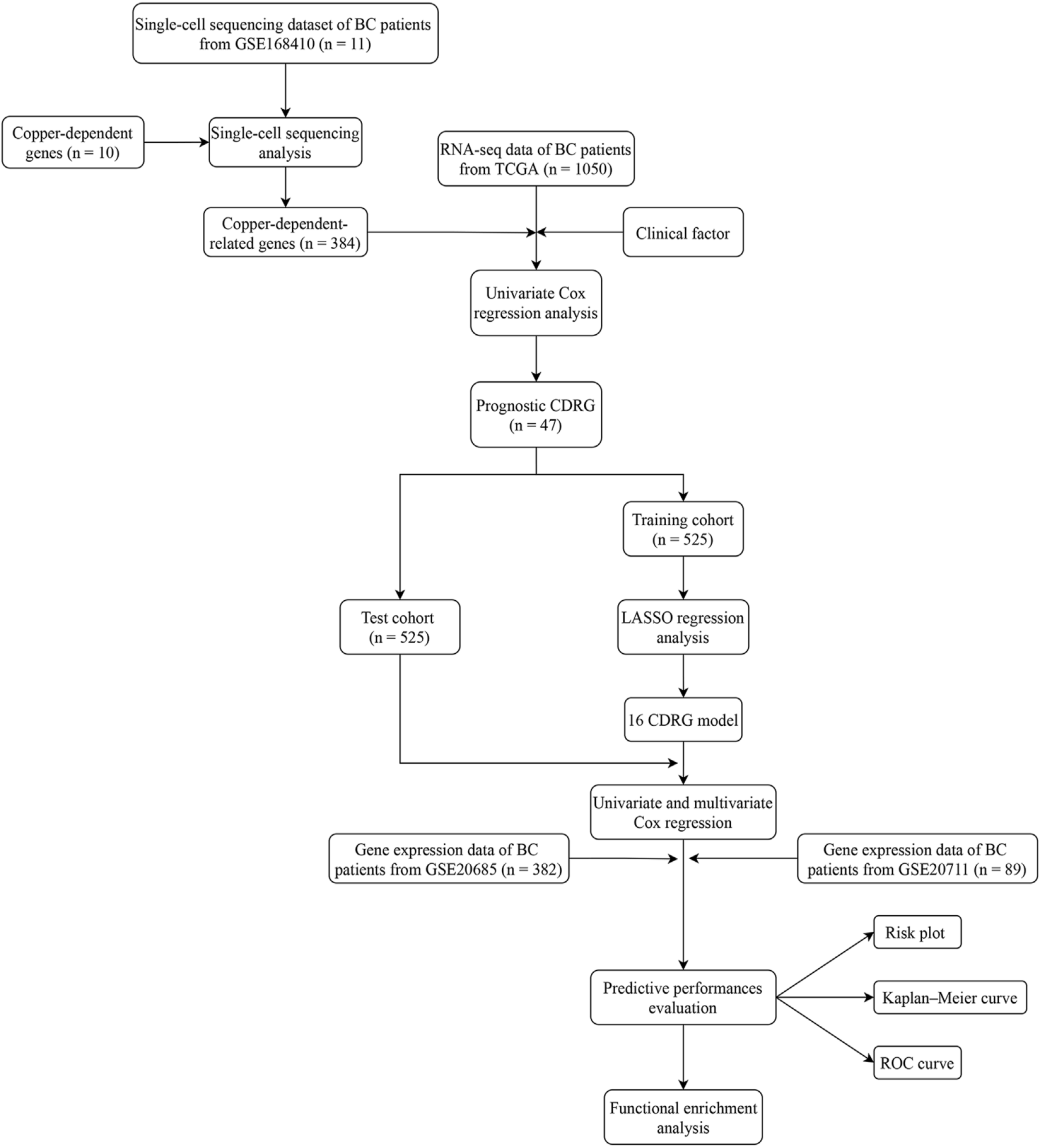
The flow chart of data collection and analysis in this study.

A flow chart of our work was shown in Figure 1.

### Data collection

The “TCGAbiolinks” R package was used to download TCGA data (TCGA-BRCA; URL: https://portal.gdc.cancer.gov/; data: 31 May 2022; Version: v33.1). The TCGA database was used to download transcriptome and clinical data. The workflow type we used was Counts. 10 copper-dependent genes (Negative hits: *MTF1*, *GLS*, *CDKN2A*; Positive hits: *FDX1*, *LIAS*, *LIPT1*, *DLD*, *DLAT*, *PDHA1*, *PDHB*) were obtained from the study by Tsvetkov et al. (2022). We downloaded the BC single-cell sequencing dataset GSE168410 (Kester et al., 2022) from the GEO database (https://www.ncbi.nlm.nih.gov/geo/). We also downloaded two databases GSE20685 (Kao et al., 2011) and GSE20711 (Dedeurwaerder et al., 2011) from the GEO database as the external validation cohorts to validate our model.

### Data processing of the GSE168410

First, we performed quality control on the data. As patient10 had less single-cell sequencing data, we excluded patient10 and used single-cell sequencing data from the remaining 11 patients for subsequent analysis. Cells with fewer than 5% mitochondrial genes and a total amount of genes over 300 were kept. Genes expressed in at least three cells were kept. Stacked histograms were used to show the proportion of cells in each sample. We screened out the 6,000 most fluctuating genes according to their fluctuating degrees in all samples. We used the “CellCycleScoring” function to judge the selected cell cycle and used the “ScaleData” function to eliminate the effect caused by the cell cycle. The LogNormalize method was used to normalize and integrate the samples. After the data was corrected, principal component analysis was used for dimensionality reduction of the data, and TSNE was used for cluster analysis. We annotated cell types using the “SingleR” package. We download the singler database, load “ref_Human_all.Rdata” into the environment, and define cell subsets according to the singler algorithm. And after annotating the cells, the differential genes of each cluster were obtained by FindAllMarkers detection. After importing copper-dependent genes, the proportion of them in each cell was calculated by the PercentageFeatureSet function. According to the median ratio of copper-dependent genes, we divided the cells into low_cuproptosis and high_cuproptosis cells. Then, we use the FindMarkers function to find the differential genes of low_cuproptosis and high_cuproptosis cells, and filter the genes to screen out the genes whose *p*-value is less than 0.05. We defined these genes as copper-dependence-related genes (CDRG).

### Data processing of the TCGA

First, the data downloaded were preprocessed and combined using the Perl language to access the count file. The gene symbol was also transformed with Perl. Then, the corresponding gene expression was acquired by matching the transcriptome data from TCGA with CDRG. We excluded patients with incomplete clinical data and those with 0 days of follow-up. We then performed a subgroup analysis of the patients. We matched the CDRG expression data with the survival data, performed a univariate COX analysis, and screened out genes with a *p*-value less than 0.05, which were prognostically significant genes. The forest diagram was used to show the prognostic genes.

### Construction of the prognostic model and nomogram

We used the “caret” package to randomly split the matched cohort into a training cohort and a test cohort in a 1:1 ratio. Subsequently, the prognostic CDRG were selected by the least absolute and selection operator (LASSO) regression. We then calculated the risk score for each patient and built a prognostic model. We divided BC patients into high- and low-risk groups based on median score. Between the two groups, we utilized a clinical correlation heatmap to analyze differences in clinical features and examined disparities in patient outcomes. The survival differences were then verified using a log-rank test. Thereafter, univariate and multivariate cox analyses were performed with the risk score and different clinical information. Subsequently, we plotted the time-dependent receiver operating characteristic (ROC) plots and calculated the area under the curve (AUC) to validate the predictive power of the constructed prognostic model. We combined clinical data and patient risk scores to construct a nomogram to further analyze the prognosis of BC patients. Finally, the nomogram’s accuracy in estimating patient outcomes was evaluated by prognostic ROC curves.

### External validation of the prognostic model

GSE20685 cohort and GSE20711cohort were selected as two external validation cohorts. In both external validation cohorts, risk scores for each sample were calculated according to the formula of the model, and patients were divided into high- and low-risk groups based on the median. Next, survival analysis was performed to determine whether there was also a difference in prognosis between the two groups in the external validation cohorts. The ROC curve was used to evaluate the accuracy of the model.

### Functional enrichment analysis

We performed the Gene Ontology (GO) analysis and the Kyoto Encyclopedia of Genes and Genomes (KEGG) pathway analysis by the “clusterProfiler” package. We also performed the gene set variation analysis (GSVA) by the “GSVA” package. The results were kept if the *p*-value < 0.05. The bar charts were used to represent the results of the analysis.

### Immunoassay and m6A analysis

To investigate the correlation between our risk model and the level of tumor infiltration, we designed the immune infiltration heatmap and the correlation map to visualize our data. The tumor infiltration methods we used were TIMER, CIBERSORT, QUANTISEQ, MCPCOUNTER, XCELL, and EPIC. The literature provided us with a list of m6a-related (Huang et al., 2020; Tang et al., 2020; Li et al., 2021) and immune checkpoint-related genes (Tian et al., 2020; Jiang et al., 2021; Shimada et al., 2021; Song et al., 2021). Boxplots were used to display the results of the analysis.

### Drug sensitivity analysis

We used the expression matrix and drug processing information from the “Cancer Genome Project” (CGP, https://www.cancerrxgene.org/) to obtain the drugs associated with the model genes using the “pRROpheticPredict” function (Geeleher et al., 2014).

## Results

### Analysis of the GEO dataset

**FIGURE 2:**
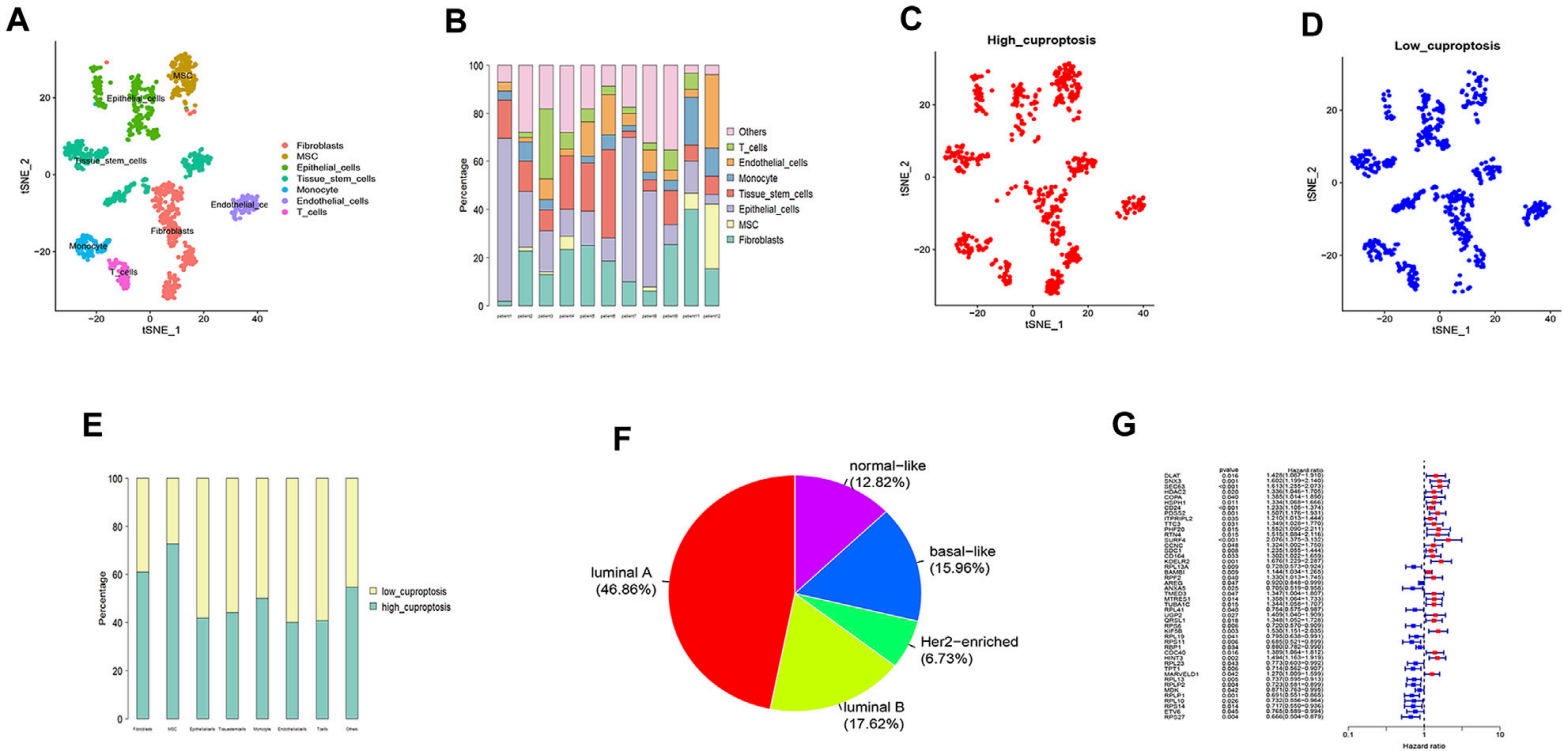
Single-cell sequencing analysis **(A,B)**, distribution of high_cuproptosis cells and low_cuproptosis cells **(C,D,E)**, subgroup analysis **(E)**, and univariate COX analysis **(G)**. **(A)** Single-cell sequencing analysis of the GSE168410 dataset (*n* = 11). According to the surface marker genes of different cell types, the cells were annotated as Fibroblasts, MSC, Epithelial_cells, Tissue_stem_cells, Monocyte, Endothelial_cells, and T_cells. **(B)** The cell ratios of every sample at the single-cell level after quality control. **(C,D)** Distribution of high_cuproptosis cells and low_cuproptosis cells. **(E)** The distribution of high_cuproptosis cells and low_cuproptosis cells in each cell cluster was relatively uniform. **(F)** The BC patients were divided into five subgroups, including luminal A (46.86%), luminal B (17.62%), Her2-enriched (6.73%), basal-like (15.96%), and normal-like (12.82%). **(G)** Univariate COX analysis of the TCGA cohort. We selected 47 genes with prognostic significance (*p* < 0.05). Blue represented low-risk CDRG and red represented high-risk CDRG.

Supplementary Figure S1A shows the amount of gene expression per cell, the ratio of mitochondrial genes and CDRG in 11 samples. Cells were evenly distributed among the 11 samples. The number of genes and their expression levels are positively correlated (Supplementary Figure S1B). We marked the top 10 genes out of 6,000 hypervariable genes in red (Supplementary Figure S1C). We then integrated the 11 samples. The result showed that the integration could be used for subsequent analysis. After PCA dimensionality reduction, using the TSNE clustering technique, we divided all cells into 13 groups and annotated all cells. According to the surface marker genes of different cell types, the cells were annotated as Fibroblasts, MSC, Epithelial_cells, Tissue_stem_cells, Monocyte, Endothelial_cells, and T_cells (Figure 2A). Figure 2B shows the ratio of different cells in each patient. Then after using the “PercentageFeatureSet” function to input 10 copper-dependent genes, the proportion of them in each cell was obtained. According to the median ratio of copper-dependent genes, we divided the cells into low_cuproptosis and high_cuproptosis cells (Figures 2C,D). The cut-off value we used was 0.04336513. We found that the distribution of low_cuproptosis cells and high_cuproptosis cells in each cell cluster was relatively uniform (Figure 2E). Finally, between the two groups, we analyzed the differentially expressed genes and identified 384 CDRG.

### Analysis of the TCGA dataset

After subgroup analysis, the patients were divided into five subgroups (Figure 2F), including luminal A (46.86%), luminal B (17.62%), Her2-enriched (6.73%), basal-like (15.96%), and normal-like (12.82%). After matching transcriptomic and clinical data of CDRG in the TCGA database, we performed independent prognostic analysis, resulting in 47 genes with prognostic significance. Figure 2G shows the CDRG associated with prognosis.

### Construction and evaluation of prognostic model

**FIGURE 3:**
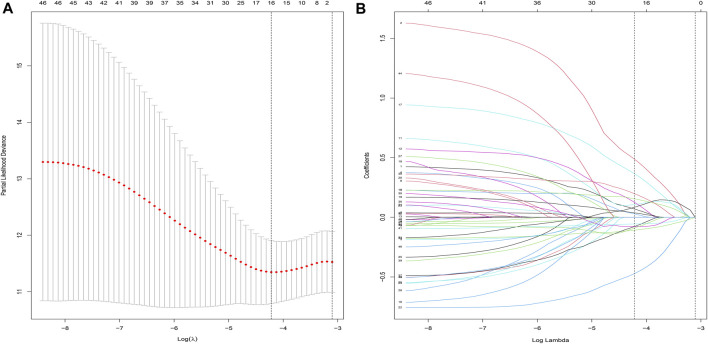
LASSO regression analysis. **(A,B)** Lasso regression was used to construct prognostic signatures in the training cohort (*n* = 525). When Lamda was 16, the curve converged.

After performing the Lasso regression analysis, we screened 16 genes from CDRG associated with prognosis in the training cohort (Figures 3A,B). At the same time, we calculated and recorded the risk score for each patient. The risk score = DLAT*0.070163 + SNX3*0.488279 + TTC3*0.028921 + PHF20*0.107077 + RTN4*0.126126 + SURF4*0.084301 + SDC1*0.155383 + KDELR2*0.366251 + BAMBI*0.037788-ANXA5*0.47211-RBP1*0.1076-TPT1*0.07754 + MARVELD1* 0.198669—MDK*0.05575-RPLP1*0.07644-ETV6*0.09076. BC patients were divided into high-risk and low-risk groups based on the median risk score (54.869275). Table 1 showed the screened genes and their coefficients.

**TABLE 1:** Genes used for model building and their Coefficients.

Gene	Coefficients
DLAT	0.070163
SNX3	0.488279
TTC3	0.028921
PHF20	0.107077
RTN4	0.126126
SURF4	0.084301
SDC1	0.155383
KDELR2	0.366251
BAMBI	0.037788
ANXA5	−0.47211
RBP1	−0.1076
TPT1	−0.07754
MARVELD1	0.198669
MDK	−0.05575
RPLP1	−0.07644
ETV6	−0.09076

Genes and their coefficients used to construct prognostic models. The risk score = DLAT*0.070163 + SNX3*0.488279 + TTC3*0.028921 + PHF20*0.107077 + RTN4* 0.126126 + SURF4*0.084301 + SDC1*0.155383 + KDELR2*0.366251 + BAMBI*0.037788-ANXA5*0.47211-RBP1*0.1076-TPT1*0.07754 + MARVELD1*0.198669–MDK*0.05575-RPLP1*0.07644-ETV6*0.09076.

**FIGURE 4:**
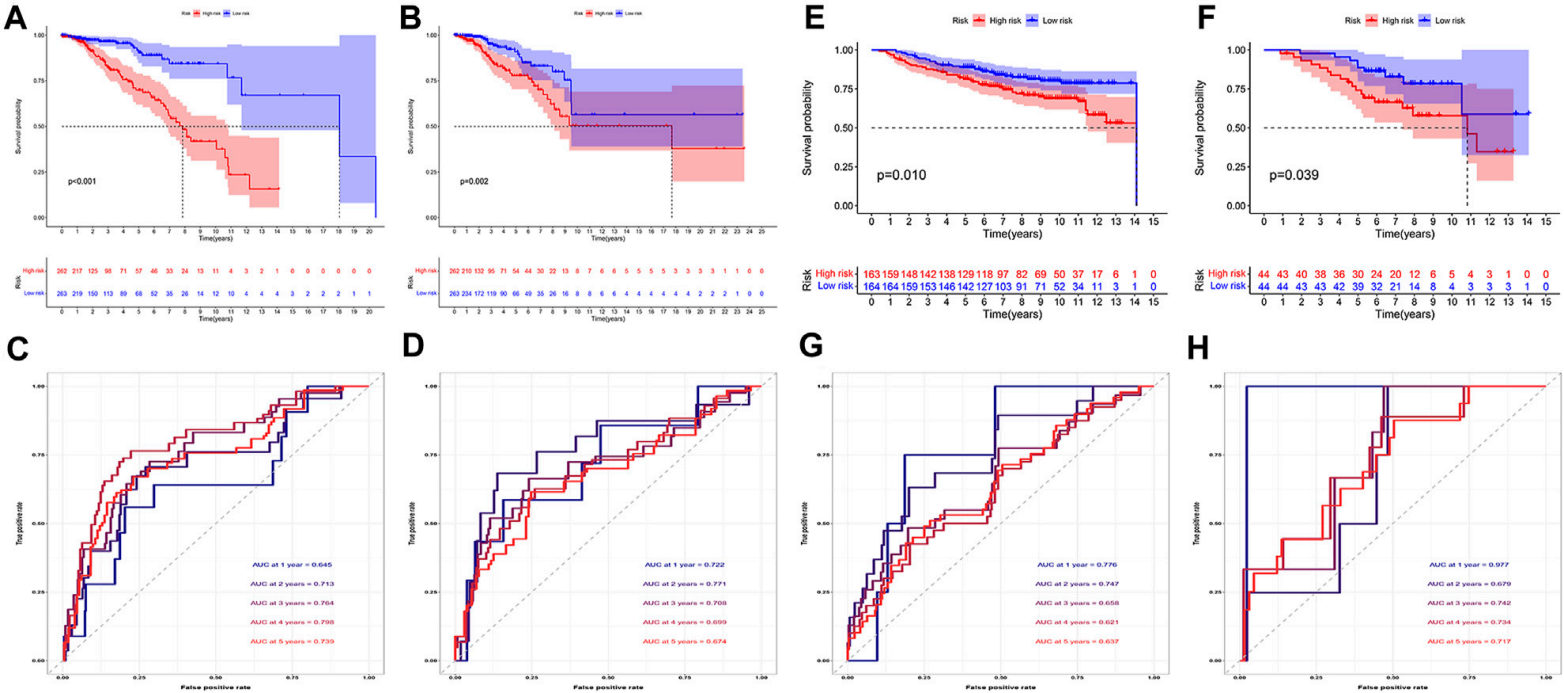
Evaluation of prognostic model. **(A,B)** In both training **(A)** and test cohorts (B, n = 525), the high-risk patients had a worse prognosis (*p* < 0.05). **(C,D)** We found that the AUC in both training **(C)** and test **(D)** cohorts were greater than or close to 0.7. **(E,F)** In both GSE20685 [**(E)**, *n* = 382] and GSE20711 [**(F)**, *n* = 89] cohorts, patients with high-risk scores had a significantly worse prognosis than those with low-risk scores (*p* < 0.05). **(G,H)** The AUC of the GSE20685 **(G)** and GSE20711 **(H)** cohorts was basically between 0.6 and 0.8.

We then evaluated the prognostic model. We performed survival analysis to explore the prognostic value of this feature. As can be seen, in both cohorts, the prognosis for patients in the high-risk group was much poorer (Figures 4A,B). The AUC at 1, 2, 3, 4, and 5 years of the training cohort were 0.645, 0.713, 0.764, 0.798, and 0.739, respectively (Figure 4C). The AUC at 1, 2, 3, 4 and 5 years of the test cohort were 0.722, 0.771, 0.708, 0.699, and 0.674, respectively (Figure 4D). Similarly, in both external validation cohorts, we also observed that patients with high-risk scores had a significantly worse prognosis than those with low-risk scores (Figures 4E,F). In order to further explore the accuracy of the prognostic model in the evaluation of the prognosis of BC patients, we conducted the ROC curve analysis in both external validation cohorts. The AUC at 1, 2, 3, 4, and 5 years of the GSE20685 cohort were 0.776, 0.747, 0.658, 0.621, and 0.637, respectively (Figure 3G). The AUC at 1, 2, 3, 4, and 5 years of the GSE20711 cohort were 0.977, 0.679, 0.742, 0.734, and 0.719, respectively (Figure 3H). The AUC in the four cohorts was greater than or near 0.7, demonstrating that the prognostic model was accurate and stable.

**FIGURE 5:**
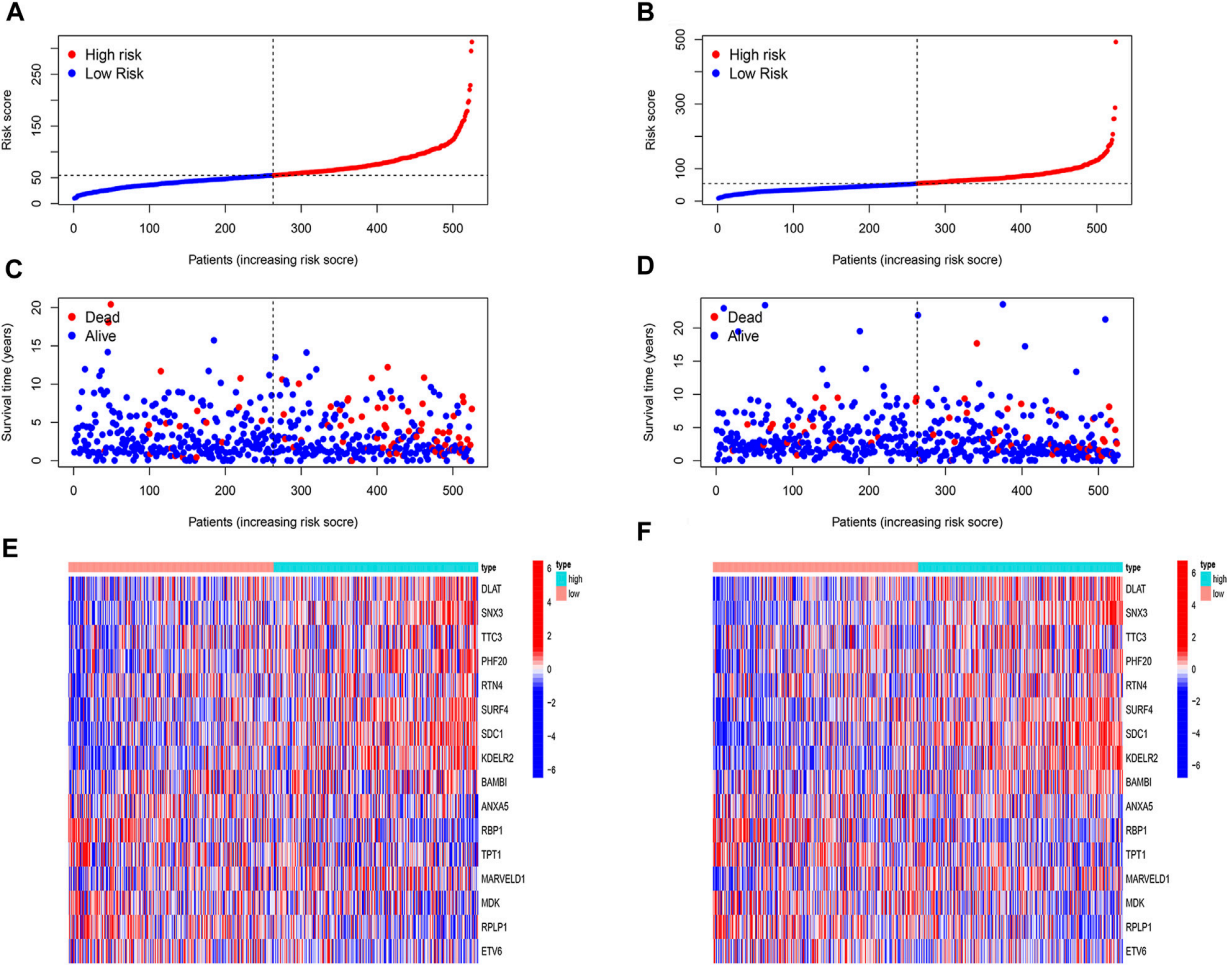
Evaluation of prognostic model. **(A,B)** The risk score of both cohorts. The patients were divided into high-risk and low-risk groups based on the median risk score. **(C,D)** The correlation of risk score and survival status of patients in both cohorts. With the increase in risk value, the proportion of BC patients who died increased. **(E,F)** Heat map of expression of 16 model genes in high-risk and low-risk in both cohorts. The genes *ANXA5*, *RBP1*, *TPT1*, *MDK*, *RPLP1*, and *ETV6* were highly expressed in the low-risk group, while *DLAT*, *SNX3*, *TTC3*, *PHF20*, *RTN4*, *SURF4*, *SDC1*, *KDELR2*, BAMBI, and *MARVELD1* were highly expressed in the high-risk group.

We then analyzed the distribution of gene expression and patient survival in the models between the high - and low-risk groups in training and test cohorts (Figures 5A,B). We found that with the increase in risk value, the proportion of BC patients who died increased (Figures 5C,D). Moreover, we found that genes *ANXA5*, *RBP1*, *TPT1*, *MDK*, *RPLP1*, and *ETV6* were highly expressed in the low-risk group, while *DLAT*, *SNX3*, *TTC3*, *PHF20*, *RTN4*, *SURF4*, *SDC1*, *KDELR2*, BAMBI, and *MARVELD1* were highly expressed in the high-risk group (Figures 5E,F).

### Exploration of independent prognostic significance of the signature

Subsequently, to see if risk score and other clinical features were independent prognostic predictors of BC, we employed univariate and multivariate COX regressions. First, univariate COX regression revealed that age (Hazard Ratio (HR) = 1.033, *p* < 0.001), stage (HR = 1.860, *p* < 0.001), and risk score (HR = 1.012, *p* < 0.001) were independent prognostic indicators of BC in the training cohort. Multivariate COX regression showed that age (HR = 1.031, *p* = 0.002, stage (HR = 2.159, *p* < 0.001), and risk score (HR = 1.012, *p* < 0.001) were independent prognostic indicators of BC. In the test cohort, univariate COX regression showed that age (HR = 1.037, *p* < 0.001), stage (HR = 2.530, *p* < 0.001), and risk score (HR = 1.011, *p* < 0.001) were independent prognostic indicators of BC. Multivariate COX regression showed that age (HR = 1.037, *p* = 0.001), stage (HR = 2.68, *p* = 0.05), and risk score (HR = 1.010, *p* = 0.037) were independent prognostic indicators of BC.

### Enrichment analysis

**FIGURE 6:**
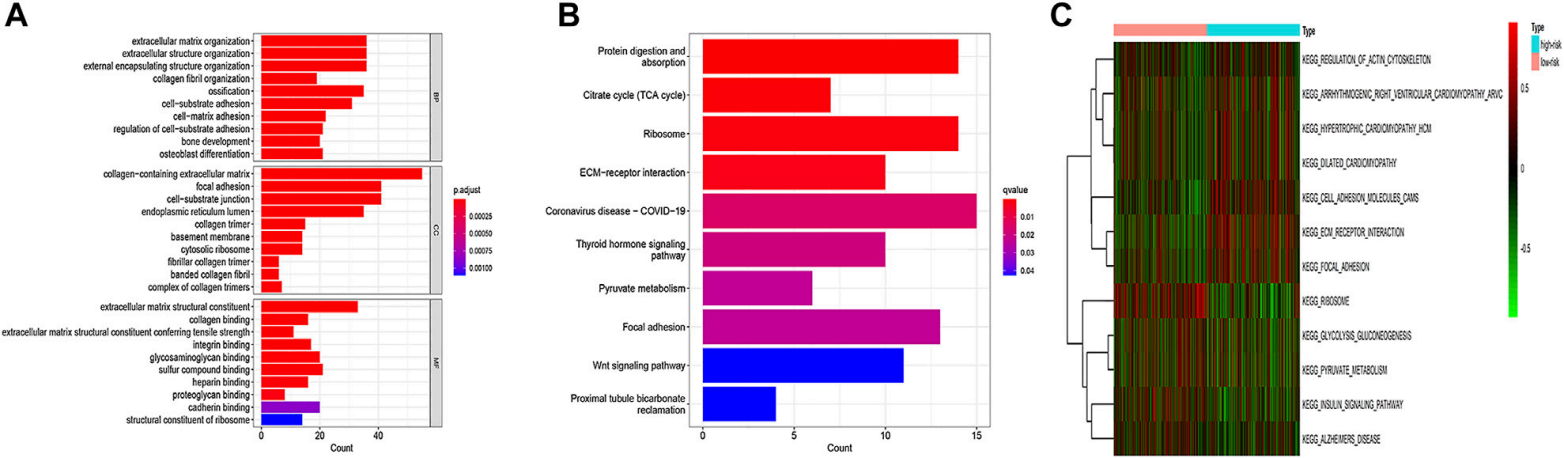
GO enrichment analysis **(A)**, KEGG enrichment analysis **(B)**, and GSVA analysis **(C)** of CDRG. **(A)** The results of GO enrichment analysis showed that these genes were mainly related to the extracellular matrix organization, extracellular framework components, and protein expression. **(B)** The results of the KEGG enrichment analysis showed that these genes were mainly related to the TCA cycle, ribosome, and protein metabolism. **(C)** The results showed that ribosome-related pathways, cell adhesion molecule-related pathways, and glycolysis and gluconeogenesis-related pathways had significant differences between high and low-risk groups.

Then, we performed the enrichment analysis. The results of GO enrichment analysis showed that these genes were mainly related to the extracellular matrix organization, extracellular framework components, and protein expression (Figure 6A). The results of the KEGG enrichment analysis showed that these genes were mainly related to the TCA cycle, ribosome, and protein metabolism (Figure 6B). GSVA analysis was used to further explore the differences in KEGG pathways involved between high and low-risk groups. The results showed that ribosome-related pathways, cell adhesion molecule-related pathways, and glycolysis and gluconeogenesis-related pathways had significant differences between high and low-risk groups (Figure 6C).

### Immunoassay and m6A analysis

**FIGURE 7:**
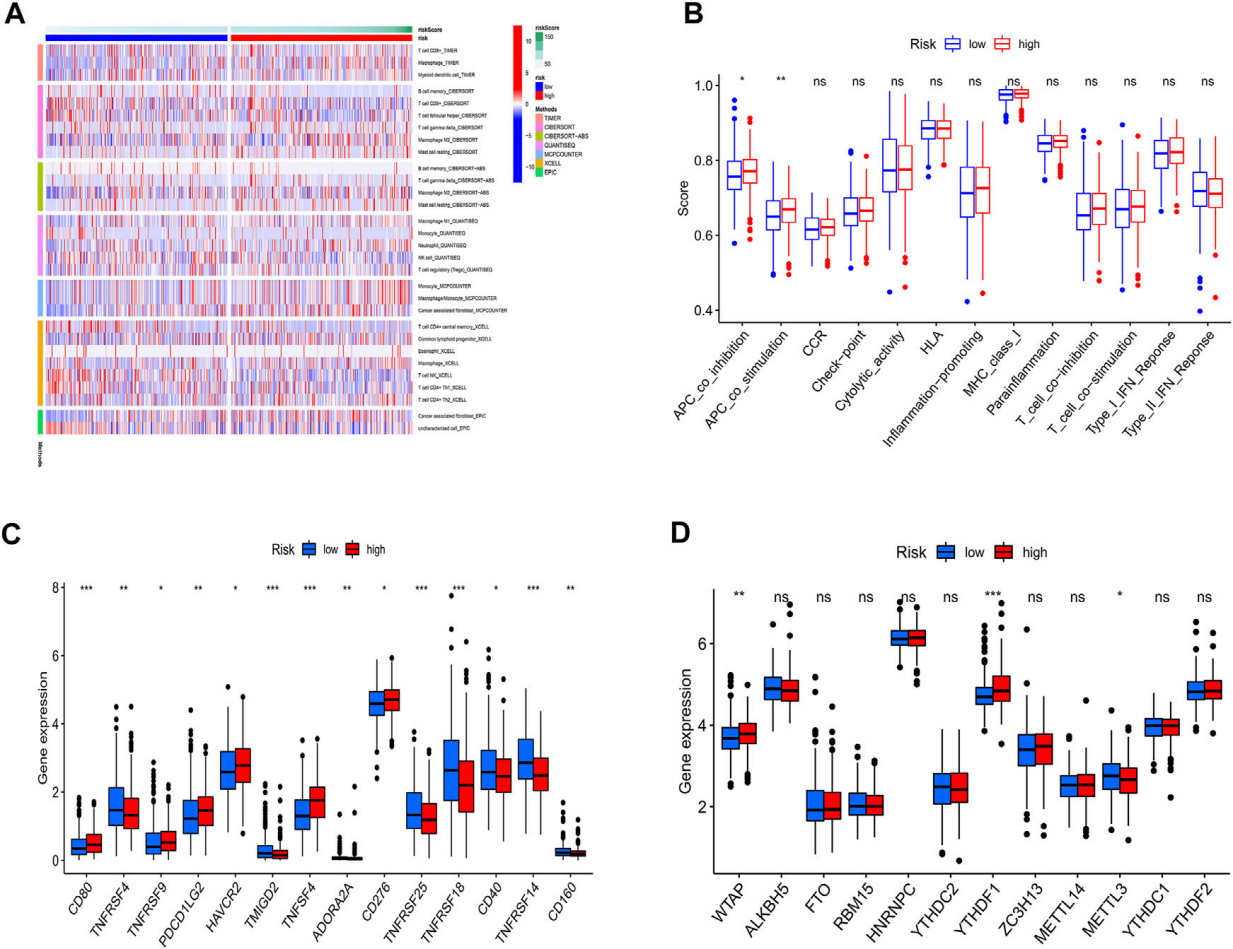
Immunoassay and m6A analysis. **(A)** Immune cell infiltration distribution. T cells, B cells, and Macrophage tended to be highly expressed mainly in the high-risk group. **(B)** Immune-related functions. The high-risk groups have a more active immune function. **(C)** The expression of immune checkpoint-related genes. The majority of immunological checkpoint genes were up-regulated. **(D)** the expression of m6A-related genes. In high-risk groups, most m6a-related genes were upregulated.

In tumor development, the immunological microenvironment is critical. T cells, B cells, and Macrophage tended to be highly expressed mainly in the high-risk group (Figure 7A).

To further understand the differences in immune microenvironments to guide immunotherapy, the immunological function of high-risk and low-risk populations was discussed. The results show that high-risk groups have a more active immune function (Figure 7B). In the high-risk group, the majority of immunological checkpoint genes were up-regulated (Figure 7C). Immune checkpoint blockade may be more beneficial to them. Meanwhile, between the two groups, Results showed that in high-risk groups, most m6a-related genes were upregulated (Figure 7D).

### Drug sensitivity analysis

**FIGURE 8:**
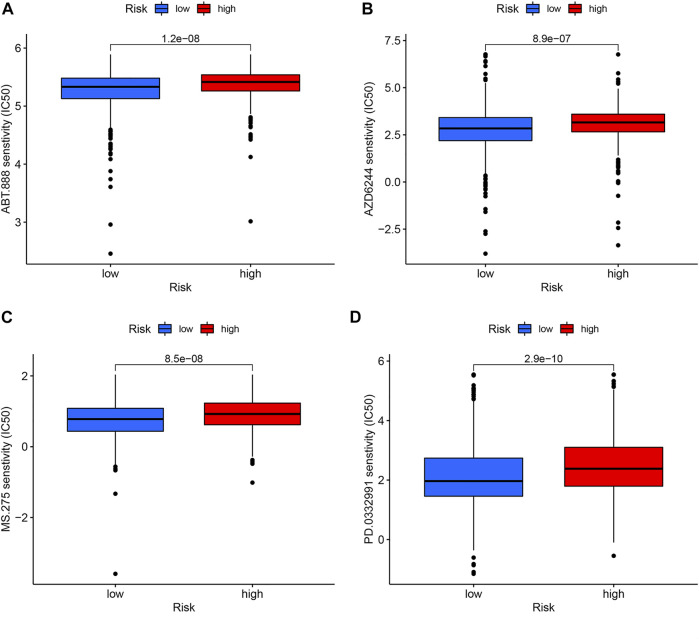
Drug sensitivity analysis. The candidates are Veliparib **(A)**, Selumetinib **(B)**, Entinostat **(C)**, and Palbociclib **(D)**.

To target treatment, drug sensitivity analyses were performed to identify drugs that were more effective in the high-risk group. Figure 8 illustrated that the candidates were Veliparib, Selumetinib, Entinostat, and Palbociclib.

### Construction of the nomogram

**FIGURE 9:**
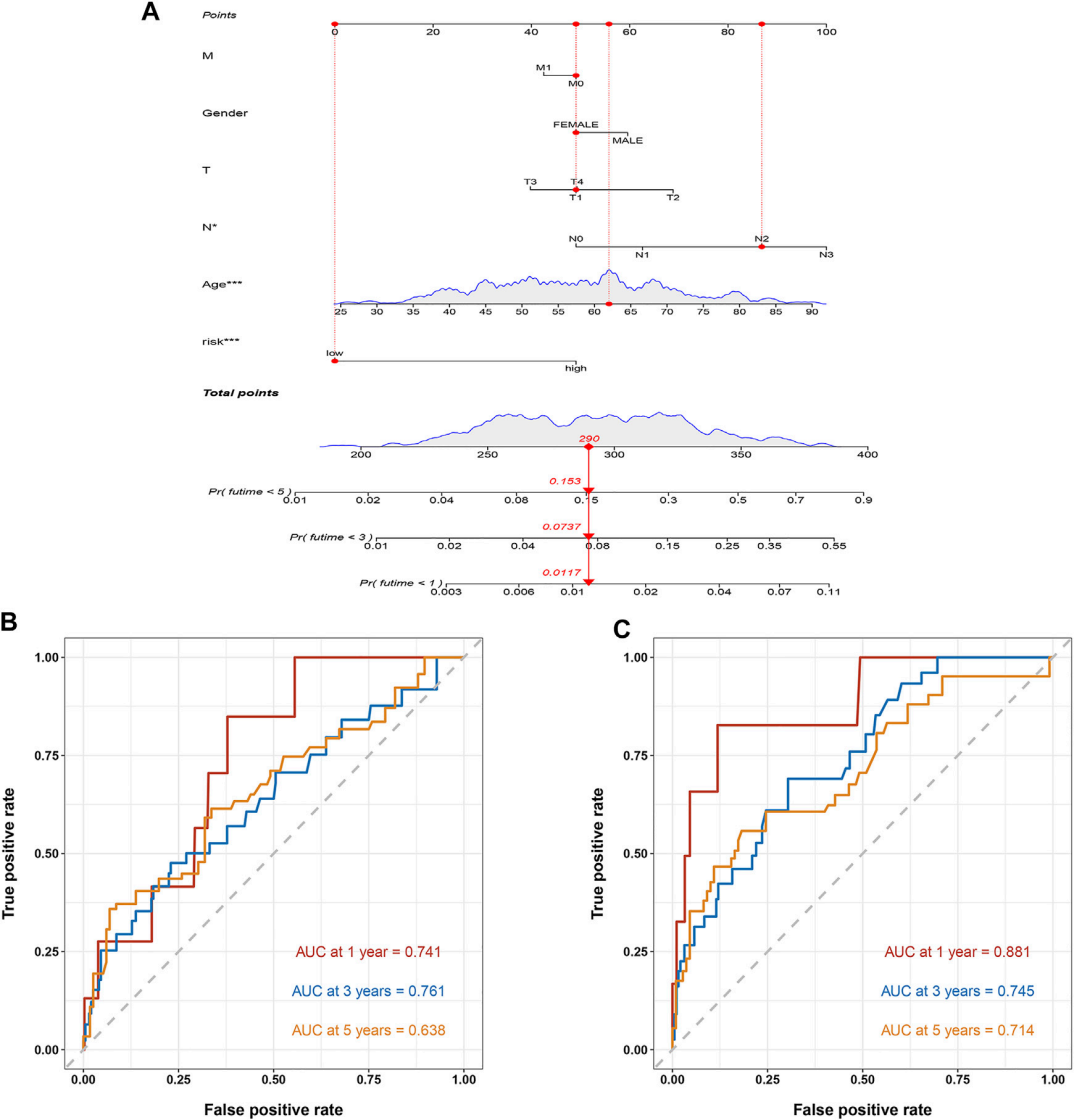
The construction of a nomogram. **(A)** The nomogram. The mortality rate of the patient in 1, 3, and 5 years was estimated to be 0.0117, 0.0737, and 0.153. **(B)** ROC curve of the nomogram in the training cohort. The AUC in 1, 3, and 5 years were 0.741, 0.761, and 0.638, respectively. **(C)** ROC curve of the nomogram in the test cohort. The AUC in 1, 3, and 5 years were 0.881, 0.745, and 0.714, respectively.

To further apply this prognostic model to BC prognostic assessment, we combined TCGA BC transcriptome data and clinical data to construct a nomogram related to the risk score. The prognostic model estimated that the 1, 3, and 5-years mortality of a BC patient was 0.0117, 0.0737, and 0.153, respectively (Figure 9A). The nomogram can better assess patient risk and guide subsequent clinical decisions. The ROC curves showed that the AUC at 1, 3 and 5 years of the training cohort were 0.741, 0.761, and 0.638, respectively (Figure 9B), and the AUC at 1, 3, and 5 years of the test cohort were 0.881, 0.745 and 0.714, respectively (Figure 9C).

## Discussion

We performed an extensive bioinformatics analysis to explore the significance of copper-dependent-related genes in BC in this study. Using the GEO and TCGA datasets, a predictive model based on copper-dependent-related genes was effectively built in this study. By calculating the risk score, patients with BC could be classified into high-risk and low-risk groups. The high-risk group showed worse outcomes in both TCGA and GEO cohorts. Besides, the ROC curves showed that this signature showed high accuracy in evaluating the prognosis of BC patients at 1, 2, 3, 4, and 5 years. In addition, we also confirmed the roles of copper-dependent-related genes in the immune microenvironment were significantly different between them, which may provide new predictors for immunotherapy in BC patients. Drug sensitivity analysis identifies more sensitive drugs for high-risk groups, which can be valuable in stratifying treatment for BC.

Female breast cancer is the most common cancer worldwide (Sung et al., 2021). Surgery, chemotherapy, radiotherapy, targeted therapy, and hormone therapy have become the main treatment strategies (Ferreira et al., 2019; Hirukawa et al., 2019; Kim et al., 2020). However, the overall prognosis of BC patients remains poor, especially for advanced patients (Tao et al., 2015). Meanwhile, despite the development of various prognostic indicators to aid clinical decision-making in BC patients, the application of predictors has been limited. The proposal of CDRG provides a novel approach to the treatment of BC.

These CDRG are significant. Copper is a cofactor for a number of essential enzymes, while copper-induced cytotoxic mechanisms can also lead to cell death (Ge et al., 2022). Studies have demonstrated that copper-induced cell death is mediated by lipid acylation of proteins that are concentrated in the TCA cycle, where lipid acylation is required for enzyme function (Solmonson and DeBerardinis, 2018; Tsvetkov et al., 2022). Copper-dependent genes are regulators of copper death (Tsvetkov et al., 2022). In this study, the results of KEGG enrichment analysis showed that the copper-dependent genes we screened were mainly associated with the TCA cycle, and GSVA analysis also showed that glycolysis and gluconeogenesis-related pathways were significantly different between high-risk and low-risk groups, confirming that our screening for copper-dependent genes is meaningful. In addition, GO enrichment analysis, KEGG enrichment analysis and GSVA analysis showed that the differential functions and pathways between the high-risk group and the low-risk group were also concentrated in extracellular matrix organization and ECM receptor interaction pathways. Therefore, we further explored the content of immune matrix components in the tumor microenvironment. The results of immune analysis confirmed that T cells, B cells and macrophages were mainly highly expressed in high-risk groups, and the high-risk groups had more active immune functions and up-regulated expression of most immune checkpoint genes. This suggests that there are more immune matrix components in the tumor microenvironment of high-risk individuals.

Sixteen genes in the prognostic model have been initially elucidated in the pathogenesis and progression of the disease. *DLAT* is involved in pyruvate metabolism and the TCA cycle (Chen et al., 2021). Goh et al. (2015) found that *DLAT* was up-regulated in gastric cancer cells and may be one of the potential drug targets in mitochondria. *SNX3* is involved in intracellular protein trafficking and acts as a key factor driving tumor progression and metastasis in triple-negative breast cancer (Cicek et al., 2022). *TTC3* is a ubiquitin E3 ligase that promotes the degradation of Akt ubiquitination and phosphorylation (Suizu et al., 2009). The study by Wu et al. (2021) constructed a 4-gene prognostic marker to evaluate and predict patients with soft tissue sarcoma, in which *TTC3* is a key molecule. *PHF20* is a multi-domain protein that regulates the activity and gene expression of P53 (Cui et al., 2012). Ma et al. (2020) identified *PHF20* as a key driver of glioblastoma malignant behavior. *RTN4* belongs to the reticulin-encoding gene family and is involved in the membrane trafficking of neuroendocrine cells (Wang et al., 2021a). *Pathak et al.* (Pathak et al., 2018) found that *RTN4* is involved in carcinogenesis, and the knockdown of *RTN4* enhanced the toxic effect of paclitaxel on cancer cells. *SURF4* encodes a conserved integral membrane protein that interacts with ER-Golgi intermediate compartment proteins (Yan et al., 2022). Kim et al. (2018) found that *SURF4* exhibited abnormal amplification and increased expression in tumor tissues. *SDC1* mediates cell binding, cell signaling, and cytoskeletal organization (Jenkins et al., 2018). Yao et al. (2019) found that *SDC1* is essential for pancreatic cancer maintenance and progression by regulating micropinocytosis. *KDELR2* is a transmembrane protein (Wang et al., 2011). Mao et al. (2020) discovered that elevated *KDELR2* expression in glioma patients was linked to a poor prognosis. *BAMBI* is a transmembrane glycoprotein whose overexpression performs an important part in the pathogenesis and development of osteosarcoma (Zhou et al., 2013). *ANXA5* is a calcium-dependent phospholipid-binding annexin (Bouter et al., 2015). Yang et al. (2021) showed that *ANXA5* is associated with chemoresistance in B-cell acute lymphoblastic leukemia. *RBP* is a carrier protein involved in the transport of retinol (Napoli, 2017). Gao et al. (2020) found that the *RBP1-CKAP4* axis is a key regulator of autophagy machinery in oral squamous cell carcinoma. *TPT1* is a protein involved in cell growth and proliferation (Bae et al., 2017). Li et al. (2019) found that the up-regulation of *TPT1* can promote the metastasis of colorectal cancer. *MARVELD1* is a nuclear protein (Wang et al., 2009). The study by Li et al. (2016) showed that gefitinib’s therapeutic efficacy in lung cancer can be improved by interfering with *MARVELD1*. *MDK* is a heparin-binding growth factor (Filippou et al., 2020). Yuan et al. (2015) found that lung cancer patients with high levels of *MDK* have a bad prognosis. *RPLP1* is a ribosomal protein (Campos et al., 2020). Xie et al. (2021) found that liver cancer patients with high levels of *RPLP1* have a bad prognosis. *ETV6* encodes an *ETS* family transcription factor and is associated with susceptibility to acute lymphoblastic leukemia (Nishii et al., 2021). Our study, combining these 16 genes to construct a prognostic model, could improve our understanding of tumor cells.

Programmed cell death is gaining increasing attention in the study of tumor therapy and the immune microenvironment (Wang et al., 2021b; Niu et al., 2022). Copper-dependent death is a newly proposed concept that occurs through the direct binding of copper to fatty acylation components of the tricarboxylic acid cycle (Tsvetkov et al., 2022). Tumor growth and metastasis have a high demand for metallic nutrients such as copper, which represents a metabolic vulnerability that can be exploited by limiting the availability of copper (Brewer, 2014; Ge et al., 2022). There are also studies that suggest copper consumption may play a role in cancer prevention (Pan et al., 2009). Tetrathiomolybdate, a less toxic copper chelator, is the primary drug used in copper depletion experiments in cancer models. It has achieved impressive results in a clinical trial (https://www.cancer.org/cancer/breast-cancer/understanding-a-breast-cancer-diagnosis/types-of- breast-cancer/triple-negative.html) in advanced breast cancer, with event-free survival rates of 90% (stage II/III) and 50% (stage IV) in patients with triple-negative breast cancer. However, studies of genes related to copper dependence in BC are lacking. For the first time, we provide the prognostic features of BC CDRG, which have crucial consequences for BC prognosis.

Research has confirmed that cancer must evade anti-tumor immune responses in order to grow gradually (Gajewski et al., 2013). Tumor immune evasion has been recognized as a hallmark of cancer progression (Batlle and Massagué, 2019). Immunotherapy for cancer has recently advanced in the treatment of advanced tumors, however, a significant proportion of patients do not respond (Pardoll, 2012; Di Giacomo et al., 2013). BC is considered to be immunologically quiescent tumors, which greatly hinders their therapeutic response to immunotherapy. However, recent studies have shown that immune infiltration in the tumor immune microenvironment plays a decisive role in predicting the prognosis of BC (Baxevanis et al., 2021). Tumor microenvironment structure in selected BC correlates with genomic profiles indicative of immune escape (Danenberg et al., 2022). The results of pre-clinical trials suggest that immunotherapy may be a new approach to the clinical management of BC (Adams et al., 2019). Therefore, it is important to understand the immune microenvironment of BC. Our study found that the high-risk group is associated with immune cell infiltration and high expression of immune checkpoint genes, and therefore the high-risk group is more likely to benefit from immunotherapy.

Immune checkpoint blockade (ICB) is expected to be a treatment modality for cancer patients (El-Khoueiry et al., 2017). However, many patients who receive immunotherapy do not respond to ICB, or patients initially respond to ICB but gradually become insensitive as the disease progresses (Pitt et al., 2016; Marin et al., 2022). M6A is considered to be the most significant and important modification of mRNA and noncoding RNA (He et al., 2019). The m6A regulators that regulate m6A modification may be involved in the growth, invasion, and metastasis of various cancers, as well as abnormal immune regulation (Gu et al., 2021; Uddin et al., 2021). Several studies have shown that the tumor microenvironment is closely linked to m6A modifications (Tang et al., 2020; Xu et al., 2020; Zhang et al., 2020). Our study found significant differences in the expression of m6a-related genes in high-risk and low-risk groups, which could help to screen patients for the benefits of immunotherapy.

Drug-resistant treatment is a major challenge in the current treatment of BC (Bai et al., 2018). Our study screens drug candidates relevant to prognostic models. In addition, between high and low-risk groups, m6A-related gene expression varies substantially, which has implications for our further breast cancer treatment.

However, our study has some limitations. Firstly, our model was constructed and validated based on retrospective data. Prospective clinical validation is needed henceforth. Secondly, The research data comes from the TCGA and GEO public databases. In the future, *in vivo* or *in vitro* basic experiments will be performed to confirm our findings, and we will further refine them in the future.

This is the first copper-dependent-related gene prognostic model of breast cancer utilizing single-cell cluster analysis that we are aware of, and it informs the study of BC programmed deaths and also contributes to the treatment of BC patients.

## Conclusion

Based on copper-dependent-related genes, the prognostic model was built in BC. We can accurately estimate the prognosis and immunological microenvironment of BC patients using this model. In addition, Our findings might lead to new approaches to BC therapy.

## Data Availability

The datasets presented in this study can be found in online repositories. The names of the repository/repositories and accession number(s) can be found in the article/Supplementary Material.

## References

[B1] Adams S. Schmid P. Rugo H. S. Winer E. P. Loirat D. Awada A. (2019). Pembrolizumab monotherapy for previously treated metastatic triple-negative breast cancer: cohort A of the phase II KEYNOTE-086 study. Ann. Oncol. 30 (3), 397–404. 10.1093/annonc/mdy517 30475950

[B2] Bae S. Y. Byun S. Bae S. H. Min D. S. Woo H. A. Lee K. (2017). TPT1 (tumor protein, translationally-controlled 1) negatively regulates autophagy through the BECN1 interactome and an MTORC1-mediated pathway. Autophagy 13 (5), 820–833. 10.1080/15548627.2017.1287650 28409693 PMC5446065

[B3] Bai X. Ni J. Beretov J. Graham P. Li Y. (2018). Cancer stem cell in breast cancer therapeutic resistance. Cancer Treat. Rev. 69, 152–163. 10.1016/j.ctrv.2018.07.004 30029203

[B4] Batlle E. Massagué J. (2019). Transforming growth factor-β signaling in immunity and cancer. Immunity 50 (4), 924–940. 10.1016/j.immuni.2019.03.024 30995507 PMC7507121

[B5] Baxevanis C. N. Fortis S. P. Perez S. A. (2021). The balance between breast cancer and the immune system: Challenges for prognosis and clinical benefit from immunotherapies. Semin. Cancer Biol. 72, 76–89. 10.1016/j.semcancer.2019.12.018 31881337

[B6] Bouter A. Carmeille R. Gounou C. Bouvet F. Degrelle S. A. Evain-Brion D. (2015). Review: Annexin-A5 and cell membrane repair. Placenta 36 (1), S43–S49. 10.1016/j.placenta.2015.01.193 25701430

[B7] Brewer G. J. (2014). The promise of copper lowering therapy with tetrathiomolybdate in the cure of cancer and in the treatment of inflammatory disease. J. Trace Elem. Med. Biol. 28 (4), 372–378. 10.1016/j.jtemb.2014.07.015 25194954

[B8] Campos R. K. Wijeratne H. R. S. Shah P. Garcia-Blanco M. A. Bradrick S. S. (2020). Ribosomal stalk proteins RPLP1 and RPLP2 promote biogenesis of flaviviral and cellular multi-pass transmembrane proteins. Nucleic Acids Res. 48 (17), 9872–9885. 10.1093/nar/gkaa717 32890404 PMC7515724

[B9] Chen S. Liu X. Peng C. Tan C. Sun H. Liu H. (2021). The phytochemical hyperforin triggers thermogenesis in adipose tissue via a Dlat-AMPK signaling axis to curb obesity. Cell Metab. 33 (3), 565–580.e7. e7. 10.1016/j.cmet.2021.02.007 33657393

[B10] Cicek E. Circir A. Oyken M. Akbulut Caliskan O. Dioken D. N. Guntekin Ergun S. (2022). EGF-SNX3-EGFR axis drives tumor progression and metastasis in triple-negative breast cancers. Oncogene 41 (2), 220–232. 10.1038/s41388-021-02086-9 34718348 PMC8883427

[B11] Cui G. Park S. Badeaux A. I. Kim D. Lee J. Thompson J. R. (2012). PHF20 is an effector protein of p53 double lysine methylation that stabilizes and activates p53. Nat. Struct. Mol. Biol. 19 (9), 916–924. 10.1038/nsmb.2353 22864287 PMC3454513

[B12] Danenberg E. Bardwell H. Zanotelli V. R. T. Provenzano E. Chin S. F. Rueda O. M. (2022). Breast tumor microenvironment structures are associated with genomic features and clinical outcome. Nat. Genet. 54 (5), 660–669. 10.1038/s41588-022-01041-y 35437329 PMC7612730

[B13] Dedeurwaerder S. Desmedt C. Calonne E. Singhal S. K. Haibe-Kains B. Defrance M. (2011). DNA methylation profiling reveals a predominant immune component in breast cancers. EMBO Mol. Med. 3 (12), 726–741. 10.1002/emmm.201100801 21910250 PMC3377115

[B14] Di Giacomo A. M. Calabrò L. Danielli R. Fonsatti E. Bertocci E. Pesce I. (2013). Long-term survival and immunological parameters in metastatic melanoma patients who responded to ipilimumab 10 mg/kg within an expanded access programme. Cancer Immunol. Immunother. 62 (6), 1021–1028. 10.1007/s00262-013-1418-6 23591982 PMC11029072

[B15] El-Khoueiry A. B. Sangro B. Yau T. Crocenzi T. S. Kudo M. Hsu C. (2017). Nivolumab in patients with advanced hepatocellular carcinoma (CheckMate 040): An open-label, non-comparative, phase 1/2 dose escalation and expansion trial. Lancet (London, Engl. 389 (10088), 2492–2502. 10.1016/s0140-6736(17)31046-2 PMC7539326 28434648

[B16] Faria S. S. Costantini S. de Lima V. C. C. de Andrade V. P. Rialland M. Cedric R. (2021). NLRP3 inflammasome-mediated cytokine production and pyroptosis cell death in breast cancer. J. Biomed. Sci. 28 (1), 26. 10.1186/s12929-021-00724-8 33840390 PMC8040227

[B17] Ferreira A. R. Di Meglio A. Pistilli B. Gbenou A. S. El-Mouhebb M. Dauchy S. (2019). Differential impact of endocrine therapy and chemotherapy on quality of life of breast cancer survivors: A prospective patient-reported outcomes analysis. Ann. Oncol. 30 (11), 1784–1795. 10.1093/annonc/mdz298 31591636

[B18] Filippou P. S. Karagiannis G. S. Constantinidou A. (2020). Midkine (MDK) growth factor: A key player in cancer progression and a promising therapeutic target. Oncogene 39 (10), 2040–2054. 10.1038/s41388-019-1124-8 31801970

[B19] Gajewski T. F. Schreiber H. Fu Y. X. (2013). Innate and adaptive immune cells in the tumor microenvironment. Nat. Immunol. 14 (10), 1014–1022. 10.1038/ni.2703 24048123 PMC4118725

[B20] Gao L. Wang Q. Ren W. Zheng J. Li S. Dou Z. (2020). The RBP1-CKAP4 axis activates oncogenic autophagy and promotes cancer progression in oral squamous cell carcinoma. Cell Death Dis. 11 (6), 488. 10.1038/s41419-020-2693-8 32587255 PMC7316825

[B21] Ge E. J. Bush A. I. Casini A. Cobine P. A. Cross J. R. DeNicola G. M. (2022). Connecting copper and cancer: From transition metal signalling to metalloplasia. Nat. Rev. Cancer 22 (2), 102–113. 10.1038/s41568-021-00417-2 34764459 PMC8810673

[B22] Geeleher P. Cox N. Huang R. S. (2014). pRRophetic: an R package for prediction of clinical chemotherapeutic response from tumor gene expression levels. PloS one 9 (9), e107468. 10.1371/journal.pone.0107468 25229481 PMC4167990

[B23] Goh W. Q. Ow G. S. Kuznetsov V. A. Chong S. Lim Y. P. (2015). DLAT subunit of the pyruvate dehydrogenase complex is upregulated in gastric cancer-implications in cancer therapy. Am. J. Transl. Res. 7 (6), 1140–1151. 26279757 PMC4532746

[B24] Gu Y. Wu X. Zhang J. Fang Y. Pan Y. Shu Y. (2021). The evolving landscape of N(6)-methyladenosine modification in the tumor microenvironment. Mol. Ther. 29 (5), 1703–1715. 10.1016/j.ymthe.2021.04.009 33839323 PMC8116604

[B25] Harbeck N. Penault-Llorca F. Cortes J. Gnant M. Houssami N. Poortmans P. (2019). Breast cancer. Nat. Rev. Dis. Prim. 5 (1), 66. 10.1038/s41572-019-0111-2 31548545

[B26] Hassannia B. Vandenabeele P. Vanden Berghe T. (2019). Targeting ferroptosis to iron out cancer. Cancer Cell 35 (6), 830–849. 10.1016/j.ccell.2019.04.002 31105042

[B27] He L. Li H. Wu A. Peng Y. Shu G. Yin G. (2019). Functions of N6-methyladenosine and its role in cancer. Mol. Cancer 18 (1), 176. 10.1186/s12943-019-1109-9 31801551 PMC6892141

[B28] Hirukawa A. Singh S. Wang J. Rennhack J. P. Swiatnicki M. Sanguin-Gendreau V. (2019). Reduction of global H3K27me(3) enhances HER2/ErbB2 targeted therapy. Cell Rep. 29 (2), 249–257. e8. 10.1016/j.celrep.2019.08.105 31597089

[B29] Huang G. Z. Wu Q. Q. Zheng Z. N. Shao T. R. Chen Y. C. Zeng W. S. (2020). M6A-related bioinformatics analysis reveals that HNRNPC facilitates progression of OSCC via EMT. Aging 12 (12), 11667–11684. 10.18632/aging.103333 32526707 PMC7343469

[B30] Hwang B. Lee J. H. Bang D. (2018). Single-cell RNA sequencing technologies and bioinformatics pipelines. Exp. Mol. Med. 50 (8), 96–14. 10.1038/s12276-018-0071-8 PMC6082860 30089861

[B31] Jenkins L. M. Horst B. Lancaster C. L. Mythreye K. (2018). Dually modified transmembrane proteoglycans in development and disease. Cytokine Growth Factor Rev. 39, 124–136. 10.1016/j.cytogfr.2017.12.003 29291930 PMC5866756

[B32] Jiang F. Wang X. Y. Wang M. Y. Mao Y. Miao X. L. Wu C. Y. (2021). An immune checkpoint-related gene signature for predicting survival of pediatric acute myeloid leukemia. J. Oncol. 2021, 5550116. 10.1155/2021/5550116 33986802 PMC8079183

[B33] Jin J. Ma M. Shi S. Wang J. Xiao P. Yu H. F. (2022). Copper enhances genotoxic drug resistance via ATOX1 activated DNA damage repair. Cancer Lett. 536, 215651. 10.1016/j.canlet.2022.215651 35315340

[B34] Kao K. J. Chang K. M. Hsu H. C. Huang A. T. (2011). Correlation of microarray-based breast cancer molecular subtypes and clinical outcomes: Implications for treatment optimization. BMC cancer 11, 143. 10.1186/1471-2407-11-143 21501481 PMC3094326

[B35] Kester L. Seinstra D. van Rossum A. G. J. Vennin C. Hoogstraat M. van der Velden D. (2022). Differential survival and therapy benefit of patients with breast cancer are characterized by distinct epithelial and immune cell microenvironments. Clin. Cancer Res. 28 (5), 960–971. 10.1158/1078-0432.Ccr-21-1442 34965952 PMC9377758

[B36] Kim J. Hong C. M. Park S. M. Shin D. H. Kim J. Y. Kwon S. M. (2018). SURF4 has oncogenic potential in NIH3T3 cells. Biochem. Biophys. Res. Commun. 502 (1), 43–47. 10.1016/j.bbrc.2018.05.116 29777698

[B37] Kim K. W. Jeong J. U. Lee K. H. Uong T. N. T. Rhee J. H. Ahn S. J. (2020). Combined NK cell therapy and radiation therapy exhibit long-term therapeutic and antimetastatic effects in a human triple negative breast cancer model. Int. J. Radiat. Oncol. Biol. Phys. 108 (1), 115–125. 10.1016/j.ijrobp.2019.09.041 31605787

[B38] Krop I. Ismaila N. Andre F. Bast R. C. Barlow W. Collyar D. E. (2017). Use of biomarkers to guide decisions on adjuvant systemic therapy for women with early-stage invasive breast cancer: American society of clinical oncology clinical practice guideline focused update. J. Clin. Oncol. 35 (24), 2838–2847. 10.1200/jco.2017.74.0472 28692382 PMC5846188

[B39] Li J. Yan H. Zhao L. Jia W. Yang H. Liu L. (2016). Inhibition of SREBP increases gefitinib sensitivity in non-small cell lung cancer cells. Oncotarget 7 (32), 52392–52403. 10.18632/oncotarget.10721 27447558 PMC5239560

[B40] Li R. Zhu H. Yang D. Xia J. Zheng Z. (2019). Long noncoding RNA lncBRM promotes proliferation and invasion of colorectal cancer by sponging miR-204-3p and upregulating TPT1. Biochem. Biophys. Res. Commun. 508 (4), 1259–1263. 10.1016/j.bbrc.2018.12.053 30563768

[B41] Li Y. Qi D. Zhu B. Ye X. (2021). Analysis of m6A RNA methylation-related genes in liver hepatocellular carcinoma and their correlation with survival. Int. J. Mol. Sci. 22 (3), 1474. 10.3390/ijms22031474 33540684 PMC7867233

[B42] Ma Q. Long W. Xing C. Jiang C. Su J. Wang H. Y. (2020). PHF20 promotes glioblastoma cell malignancies through a WISP1/BGN-dependent pathway. Front. Oncol. 10, 573318. 10.3389/fonc.2020.573318 33117706 PMC7574681

[B43] Mao H. Nian J. Wang Z. Li X. Huang C. (2020). KDELR2 is an unfavorable prognostic biomarker and regulates CCND1 to promote tumor progression in glioma. Pathol. Res. Pract. 216 (7), 152996. 10.1016/j.prp.2020.152996 32534703

[B44] Marin J. J. G. Romero M. R. Herraez E. Asensio M. Ortiz-Rivero S. Sanchez-Martin A. (2022). Mechanisms of pharmacoresistance in hepatocellular carcinoma: New drugs but old problems. Semin. Liver Dis. 42 (1), 87–103. 10.1055/s-0041-1735631 34544160

[B45] Maung M. T. Carlson A. Olea-Flores M. Elkhadragy L. Schachtschneider K. M. Navarro-Tito N. (2021). The molecular and cellular basis of copper dysregulation and its relationship with human pathologies. FASEB J. 35 (9), e21810. 10.1096/fj.202100273RR 34390520

[B46] Napoli J. L. (2017). Cellular retinoid binding-proteins, CRBP, CRABP, FABP5: Effects on retinoid metabolism, function and related diseases. Pharmacol. Ther. 173, 19–33. 10.1016/j.pharmthera.2017.01.004 28132904 PMC5408321

[B47] Nishii R. Baskin-Doerfler R. Yang W. Oak N. Zhao X. Yang W. (2021). Molecular basis of ETV6-mediated predisposition to childhood acute lymphoblastic leukemia. Blood 137 (3), 364–373. 10.1182/blood.2020006164 32693409 PMC7819760

[B48] Niu X. Chen L. Li Y. Hu Z. He F. (2022). Ferroptosis, necroptosis, and pyroptosis in the tumor microenvironment: Perspectives for immunotherapy of SCLC. Semin. Cancer Biol. S1044-579X (22), 00065–00067. 10.1016/j.semcancer.2022.03.009 35288298

[B49] Pan Q. Rosenthal D. T. Bao L. Kleer C. G. Merajver S. D. (2009). Antiangiogenic tetrathiomolybdate protects against Her2/neu-induced breast carcinoma by hypoplastic remodeling of the mammary gland. Clin. Cancer Res. 15 (23), 7441–7446. 10.1158/1078-0432.Ccr-09-1361 19934283 PMC3471244

[B50] Pardoll D. M. (2012). The blockade of immune checkpoints in cancer immunotherapy. Nat. Rev. Cancer 12 (4), 252–264. 10.1038/nrc3239 22437870 PMC4856023

[B51] Pathak G. P. Shah R. Kennedy B. E. Murphy J. P. Clements D. Konda P. (2018). RTN4 knockdown dysregulates the AKT pathway, destabilizes the cytoskeleton, and enhances paclitaxel-induced cytotoxicity in cancers. Mol. Ther. 26 (8), 2019–2033. 10.1016/j.ymthe.2018.05.026 30078441 PMC6094397

[B52] Pitt J. M. Vétizou M. Daillère R. Roberti M. P. Yamazaki T. Routy B. (2016). Resistance mechanisms to immune-checkpoint blockade in cancer: Tumor-intrinsic and -extrinsic factors. Immunity 44 (6), 1255–1269. 10.1016/j.immuni.2016.06.001 27332730

[B53] Rainey L. Eriksson M. Trinh T. Czene K. Broeders M. J. M. van der Waal D. (2020). The impact of alcohol consumption and physical activity on breast cancer: The role of breast cancer risk. Int. J. Cancer 147 (4), 931–939. 10.1002/ijc.32846 31863475 PMC7383781

[B54] Shanbhag V. C. Gudekar N. Jasmer K. Papageorgiou C. Singh K. Petris M. J. (2021). Copper metabolism as a unique vulnerability in cancer. Biochim. Biophys. Acta. Mol. Cell Res. 1868 (2), 118893. 10.1016/j.bbamcr.2020.118893 33091507 PMC7779655

[B55] Shimada K. Yoshida K. Suzuki Y. Iriyama C. Inoue Y. Sanada M. (2021). Frequent genetic alterations in immune checkpoint-related genes in intravascular large B-cell lymphoma. Blood 137 (11), 1491–1502. 10.1182/blood.2020007245 33512416 PMC7976508

[B56] Solmonson A. DeBerardinis R. J. (2018). Lipoic acid metabolism and mitochondrial redox regulation. J. Biol. Chem. 293 (20), 7522–7530. 10.1074/jbc.TM117.000259 29191830 PMC5961061

[B57] Song D. Tian J. Han X. Li X. (2021). A model of seven immune checkpoint-related genes predicting overall survival for head and neck squamous cell carcinoma. Eur. Arch. Otorhinolaryngol. 278 (9), 3467–3477. 10.1007/s00405-020-06540-4 33449165

[B58] Suizu F. Hiramuki Y. Okumura F. Matsuda M. Okumura A. J. Hirata N. (2009). The E3 ligase TTC3 facilitates ubiquitination and degradation of phosphorylated Akt. Dev. Cell 17 (6), 800–810. 10.1016/j.devcel.2009.09.007 20059950

[B59] Sung H. Ferlay J. Siegel R. L. Laversanne M. Soerjomataram I. Jemal A. (2021). Global cancer statistics 2020: GLOBOCAN estimates of incidence and mortality worldwide for 36 cancers in 185 countries. Ca. Cancer J. Clin. 71 (3), 209–249. 10.3322/caac.21660 33538338

[B60] Tang R. Zhang Y. Liang C. Xu J. Meng Q. Hua J. (2020). The role of m6A-related genes in the prognosis and immune microenvironment of pancreatic adenocarcinoma. PeerJ 8, e9602. 10.7717/peerj.9602 33062408 PMC7528816

[B61] Tao Z. Shi A. Lu C. Song T. Zhang Z. Zhao J. (2015). Breast cancer: Epidemiology and etiology. Cell biochem. Biophys. 72 (2), 333–338. 10.1007/s12013-014-0459-6 25543329

[B62] Tian M. Yang J. Han J. He J. Liao W. (2020). A novel immune checkpoint-related seven-gene signature for predicting prognosis and immunotherapy response in melanoma. Int. Immunopharmacol. 87, 106821. 10.1016/j.intimp.2020.106821 32731180

[B63] Treutlein B. Lee Q. Y. Camp J. G. Mall M. Koh W. Shariati S. A. (2016). Dissecting direct reprogramming from fibroblast to neuron using single-cell RNA-seq. Nature 534 (7607), 391–395. 10.1038/nature18323 27281220 PMC4928860

[B64] Tsvetkov P. Coy S. Petrova B. Dreishpoon M. Verma A. Abdusamad M. (2022). Copper induces cell death by targeting lipoylated TCA cycle proteins. Sci. (New York, N.Y.) 375 (6586), 1254–1261. 10.1126/science.abf0529 PMC9273333 35298263

[B65] Uddin M. B. Wang Z. Yang C. (2021). The m(6)A RNA methylation regulates oncogenic signaling pathways driving cell malignant transformation and carcinogenesis. Mol. Cancer 20 (1), 61. 10.1186/s12943-021-01356-0 33814008 PMC8019509

[B66] Voli F. Valli E. Lerra L. Kimpton K. Saletta F. Giorgi F. M. (2020). Intratumoral copper modulates PD-L1 expression and influences tumor immune evasion. Cancer Res. 80 (19), 4129–4144. 10.1158/0008-5472.Can-20-0471 32816860

[B67] Wang H. Lin D. Yu Q. Li Z. Lenahan C. Dong Y. (2021). A promising future of ferroptosis in tumor therapy. Front. Cell Dev. Biol. 9, 629150. 10.3389/fcell.2021.629150 34178977 PMC8219969

[B68] Wang J. Miao Y. Wicklein R. Sun Z. Wang J. Jude K. M. (2021). RTN4/NoGo-receptor binding to Bai adhesion-GPCRs regulates neuronal development. Cell 184 (24), 5869–5885.e25. e25. 10.1016/j.cell.2021.10.016 34758294 PMC8620742

[B69] Wang P. Li B. Zhou L. Fei E. Wang G. (2011). The KDEL receptor induces autophagy to promote the clearance of neurodegenerative disease-related proteins. Neuroscience 190, 43–55. 10.1016/j.neuroscience.2011.06.008 21684323

[B70] Wang S. Li Y. Han F. Hu J. Yue L. Yu Y. (2009). Identification and characterization of MARVELD1, a novel nuclear protein that is down-regulated in multiple cancers and silenced by DNA methylation. Cancer Lett. 282 (1), 77–86. 10.1016/j.canlet.2009.03.008 19364627

[B71] Wu C. Gong S. Osterhoff G. Schopow N. (2021). A novel four-gene prognostic signature for prediction of survival in patients with soft tissue sarcoma. Cancers 13 (22), 5837. 10.3390/cancers13225837 34830998 PMC8616347

[B72] Xie C. Cao K. Peng D. Qin L. (2021). RPLP1 is highly expressed in hepatocellular carcinoma tissues and promotes proliferation, invasion and migration of human hepatocellular carcinoma Hep3b cells. Exp. Ther. Med. 22 (1), 752. 10.3892/etm.2021.10184 34035849 PMC8135124

[B73] Xie F. Peng F. (2021). Reduction in copper uptake and inhibition of prostate cancer cell proliferation by novel steroid-based compounds. Anticancer Res. 41 (12), 5953–5958. 10.21873/anticanres.15414 34848449

[B74] Xu F. Chen J. X. Yang X. B. Hong X. B. Li Z. X. Lin L. (2020). Analysis of lung adenocarcinoma subtypes based on immune signatures identifies clinical implications for cancer therapy. Mol. Ther. Oncolytics 17, 241–249. 10.1016/j.omto.2020.03.021 32346613 PMC7183104

[B75] Yan R. Chen K. Wang B. Xu K. (2022). SURF4-induced tubular ERGIC selectively expedites ER-to-Golgi transport. Dev. Cell 57 (4), 512–525.e8. e8. 10.1016/j.devcel.2021.12.018 35051356 PMC8891076

[B76] Yang J. Liu P. Ma D. Zhao P. Zhang Y. Lu Y. (2021). Glucocorticoid resistance induced by ANXA5 overexpression in B-cell acute lymphoblastic leukemia. Pediatr. Hematol. Oncol. 38 (1), 36–48. 10.1080/08880018.2020.1810182 33231128

[B77] Yao W. Rose J. L. Wang W. Seth S. Jiang H. Taguchi A. (2019). Syndecan 1 is a critical mediator of macropinocytosis in pancreatic cancer. Nature 568 (7752), 410–414. 10.1038/s41586-019-1062-1 30918400 PMC6661074

[B78] Yu Z. Zhou R. Zhao Y. Pan Y. Liang H. Zhang J. S. (2019). Blockage of SLC31A1-dependent copper absorption increases pancreatic cancer cell autophagy to resist cell death. Cell Prolif. 52 (2), e12568. 10.1111/cpr.12568 30706544 PMC6496122

[B79] Yuan K. Chen Z. Li W. Gao C. E. Li G. Guo G. (2015). MDK protein overexpression correlates with the malignant status and prognosis of non-small cell lung cancer. Arch. Med. Res. 46 (8), 635–641. 10.1016/j.arcmed.2015.11.006 26656665

[B80] Zhang B. Wu Q. Li B. Wang D. Wang L. Zhou Y. L. (2020). m(6 A regulator-mediated methylation modification patterns and tumor microenvironment infiltration characterization in gastric cancer. Mol. Cancer 19 (1), 53. 10.1186/s12943-020-01170-0 32164750 PMC7066851

[B81] Zhou L. Park J. Jang K. Y. Park H. S. Wagle S. Yang K. H. (2013). The overexpression of BAMBI and its involvement in the growth and invasion of human osteosarcoma cells. Oncol. Rep. 30 (3), 1315–1322. 10.3892/or.2013.2569 23807684

[B82] Ziegenhain C. Vieth B. Parekh S. Reinius B. Guillaumet-Adkins A. Smets M. (2017). Comparative analysis of single-cell RNA sequencing methods. Mol. Cell 65 (4), 631–643. e4. 10.1016/j.molcel.2017.01.023 28212749

