# Supplementary material for: Construction of a prognostic model related to copper dependence in breast cancer by single-cell sequencing analysis
Source: Front Genet. 2022 Aug 23;13:949852. doi: 10.3389/fgene.2022.949852 (PMC9445252; doi:10.3389/fgene.2022.949852)
Supplement: Supplementary file 1 [file Table1.DOCX]

Supplementary Material

## Supplementary Figure S1


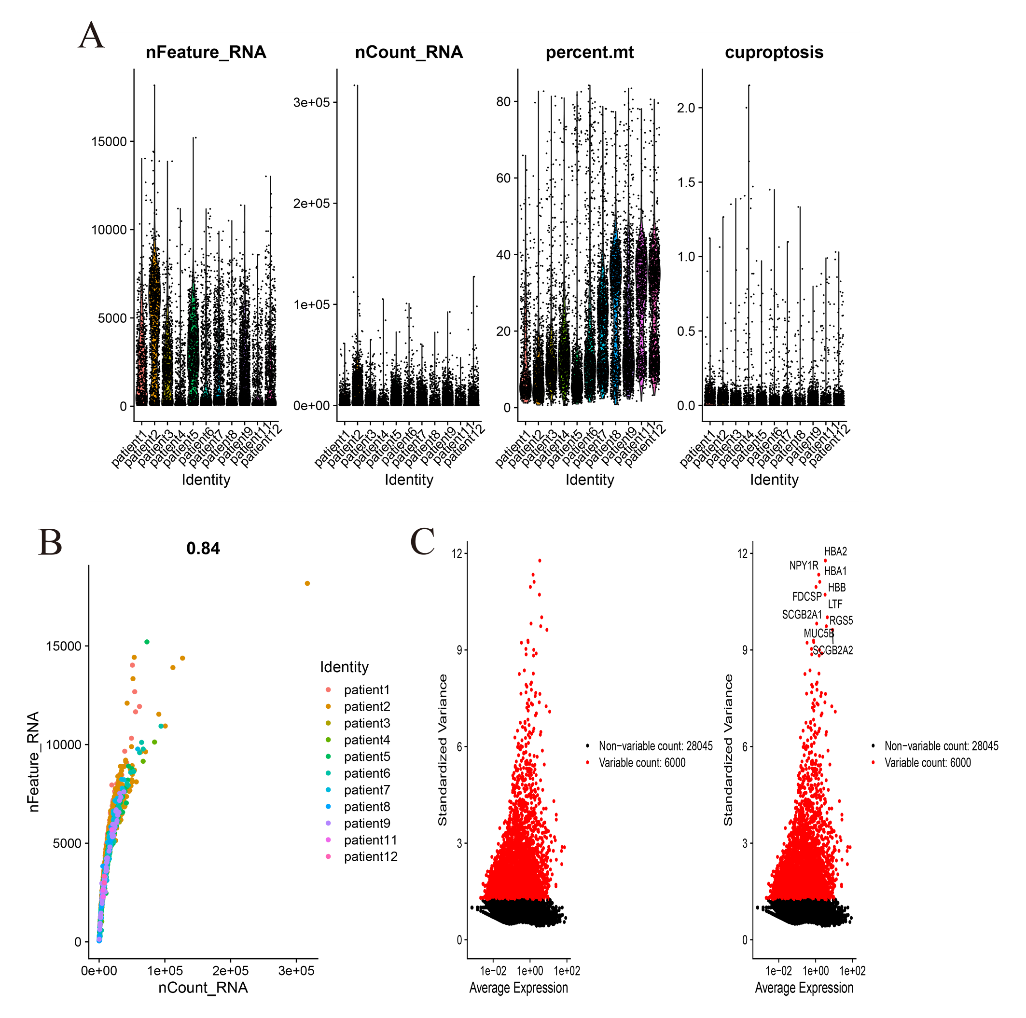


**Supplementary Figure S1**

Quality Control. (A) The amount of gene expression per cell. The ratio of mitochondrial genes. The distribution of CDRG was relatively uniform. (B) The cells were distributed evenly among the 11 samples. With a correlation coefficient of 0.8, the number of genes and their expression levels are positively correlated. (C) From all genes, we chose 6000 hypervariable genes, which were highlighted in red. We also marked the top 10genes.
