# Supplementary figures and images for: Construction of a prognostic model related to copper dependence in breast cancer by single-cell sequencing analysis
Source: Front Genet. 2022 Aug 23;13:949852. doi: 10.3389/fgene.2022.949852 (PMC9445252; doi:10.3389/fgene.2022.949852)

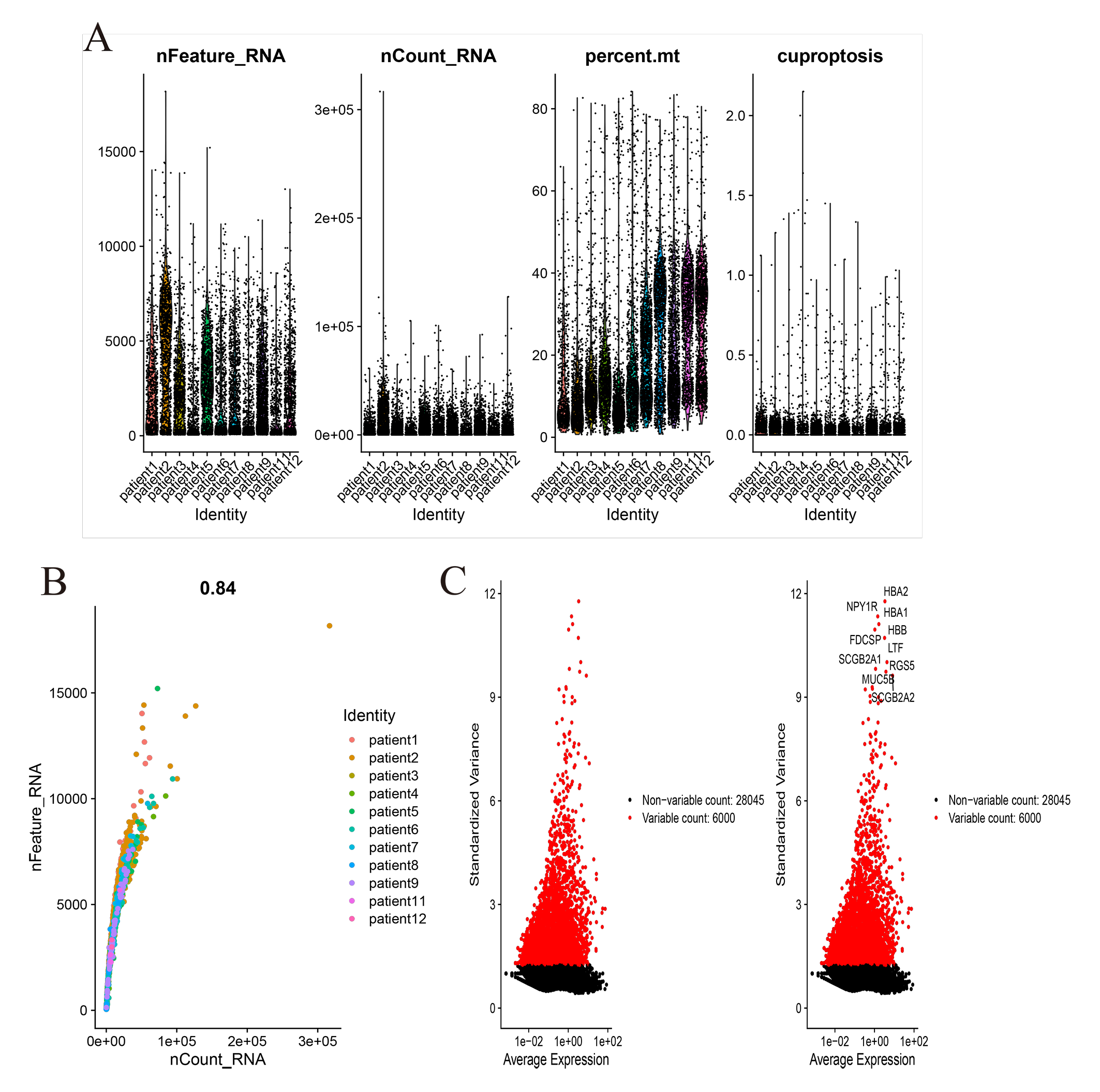

Supplement: Supplementary file 2 [file Image1.TIF]
